# Increased paclitaxel recovery from *Taxus baccata* vascular stem cells using novel in situ product recovery approaches

**DOI:** 10.1186/s40643-023-00687-8

**Published:** 2023-09-29

**Authors:** Jorge H. Santoyo-Garcia, Marissa Valdivia-Cabrera, Marisol Ochoa-Villarreal, Samuel Casasola-Zamora, Magdalena Ripoll, Ainoa Escrich, Elisabeth Moyano, Lorena Betancor, Karen J. Halliday, Gary J. Loake, Leonardo Rios-Solis

**Affiliations:** 1 Institute for Bioengineering, School of Engineering, University of Edinburgh, King’s Buildings, Edinburgh, EH9 3FB UK; 2 Centre for Engineering Biology, University of Edinburgh, King’s Buildings, Edinburgh, EH9 3BF UK; 3 Institute of Molecular Plant Sciences, School of Biological Sciences, University of Edinburgh, King’s Buildings, Edinburgh, EH9 3BF UK; 4 Green Bioactives, Douglas House, Pentland Science Park, Midlothian, EH16 0PL, UK; 5 School of Natural and Environmental Sciences, Molecular Biology and Biotechnology Division, Newcastle University, Newcastle upon Tyne, NE1 7RU UK; 6 Laboratorio de Biotecnología, Universidad ORT Uruguay, Mercedes 1237, 11100 Montevideo, Uruguay; 7 Graduate Program in Chemistry, Facultad de Química, Universidad de la República, Montevideo, Uruguay; 8 Department of Medicine and Life Sciences, Universitat Pompeu Fabra, 08003 Barcelona, Spain; 9 Department of Biochemical Engineering, The Advanced Centre for Biochemical Engineering, University College London, Gower Street, London, WC1E 6BT UK

**Keywords:** Paclitaxel, In situ product recovery, Vascular stem cells, *Taxus baccata*, Reactive oxygen species

## Abstract

In this study, several approaches were tested to optimise the production and recovery of the widely used anticancer drug Taxol^®^ (paclitaxel) from culturable vascular stem cells (VSCs) of *Taxus baccata,* which is currently used as a successful cell line for paclitaxel production. An in situ product recovery (ISPR) technique was employed, which involved combining three commercial macro-porous resin beads (HP-20, XAD7HP and HP-2MG) with batch and semi-continuous cultivations of the *T. baccata* VSCs after adding methyl jasmonate (Me-JA) as an elicitor. The optimal resin combination resulted in 234 ± 23 mg of paclitaxel per kg of fresh-weight cells, indicating a 13-fold improved yield compared to the control (with no resins) in batch cultivation. This resin treatment was further studied to evaluate the resins’ removal capacity of reactive oxygen species (ROS), which can cause poor cell growth or reduce product synthesis. It was observed that the ISPR cultivations had fourfold less intracellular ROS concentration than that of the control; thus, a reduced ROS concentration established by the resin contributed to increased paclitaxel yield, contrary to previous studies. These paclitaxel yields are the highest reported to date using VSCs, and this scalable production method could be applied for a diverse range of similar compounds utilising plant cell culture.

**Graphical Abstract:**

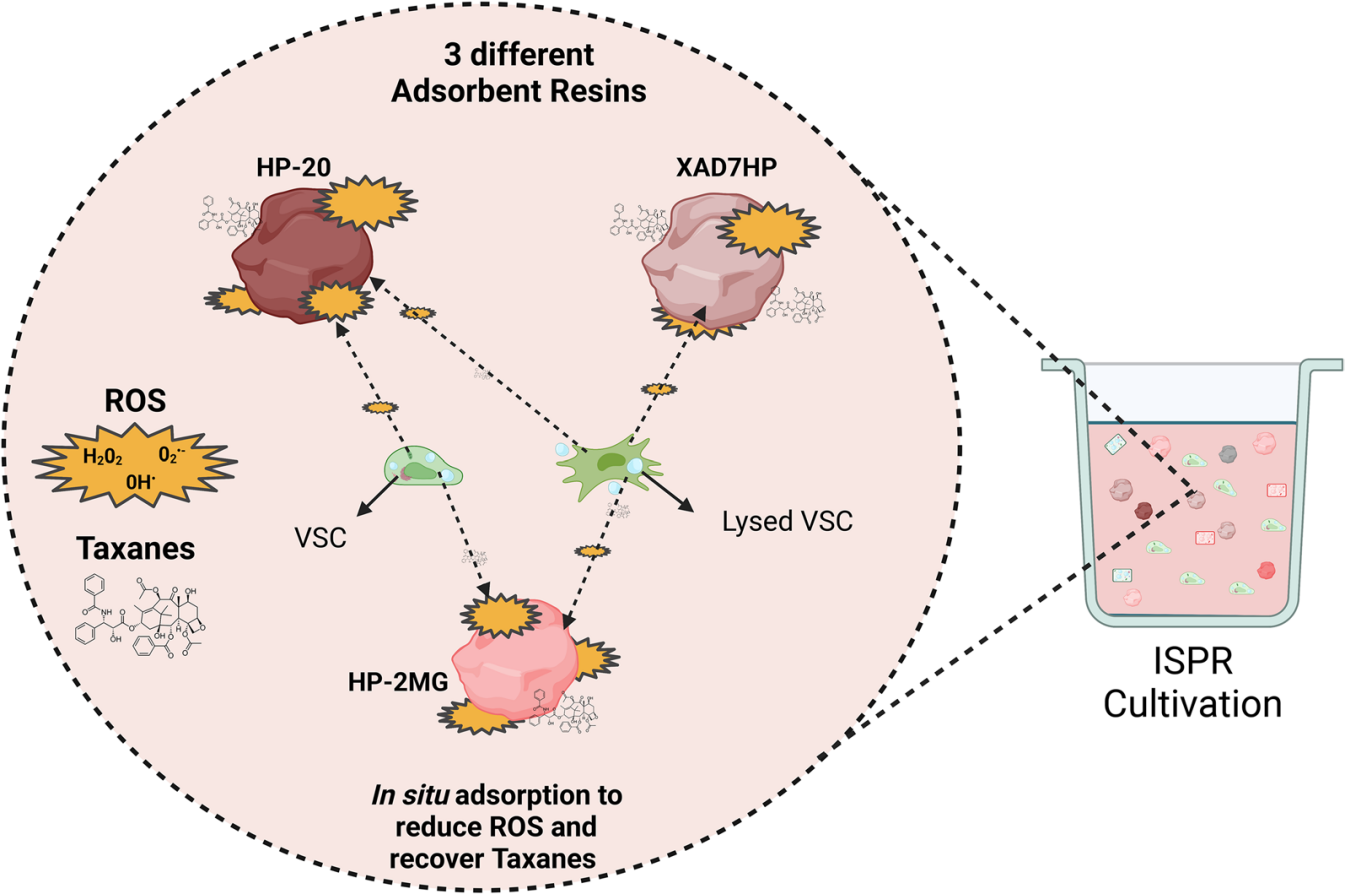

**Supplementary Information:**

The online version contains supplementary material available at 10.1186/s40643-023-00687-8.

## Introduction

Taxanes, which are a group of diterpenoids naturally synthesised by *Taxus* (yew) species, are known for their anticancer features, including the blockbuster chemotherapy drug, paclitaxel, commercially termed Taxol® (Jiang et al. 2012), a World Health Organisation (WHO) designated essential medicine. This diterpenoid is utilised in the treatment of breast, lung and ovarian cancers primarily. For example, the application of paclitaxel provides a 30% favourable response against ovarian cancer (Kampan et al. 2015). This key pharmaceutical has a unique mode of action: the inhibition of microtubule depolymerisation, thereby inhibiting mitosis provoking cell death (Naill et al. 2012; Weaver 2014). Synthetic biology techniques have been applied to produce paclitaxel entirely by heterologous expression utilising diverse microbial cell factories (Bian et al. 2017; Walls et al. 2020; Nowrouzi et al. 2020, 2022). Unfortunately, the metabolic pathway of paclitaxel has not been fully elucidated, precluding a wholly synthetic biology approach. In addition, the individual and collective optimisation of known paclitaxel enzyme function within microbial cell factories remains to be optimised (Nowrouzi and Rios-Solis 2021; Walls et al. 2022b, a). Therefore, current production of paclitaxel is mainly achieved by either plant cell–tissue cultivation or less sustainable semi-synthesis from more abundant precursors extracted by forest harvest (Kusari et al. 2014). The current demand for paclitaxel, with global sales at up to 1 billion US dollars per year (Malik et al. 2011; Precedence Research 2022), is likely to increase, as the applications for this key pharmaceutical in the treatment of atherosclerosis (Shiozaki et al. 2016), skin maladies (Montero et al. 2022) and potentially, neurodegenerative diseases (Ballatore et al. 2007) continue to expand. Therefore, security in the supply of this WHO designated essential is a pivotal issue.

### Production of paclitaxel via plant tissue cultivation from *Taxus* spp.

Currently, a key approach for the production of paclitaxel is by plant cell–tissue cultivation (Malik et al. 2011). The specific techniques vary depending on the given plant cells utilised (callus, needle cells, VSCs or protoplasts), the supporting cultivation media, in addition to the scale of production (Lee et al. 2010). One strategy for obtaining paclitaxel is through a well-established conversion of baccatin III isolated from *T. baccata* needles (Bentebibel et al. 2005). Another approach that has proven to be especially effective is the application of VSCs in different cultivation scales (Ochoa-Villarreal et al. 2016). The VSCs are isolated from the vasculature of young twigs from *Taxus* species (Lee et al. 2010; Ochoa-Villarreal et al. 2016). A key advantage for the utility of VSCs from *Taxus baccata* is their high production yield of paclitaxel relative to classical dedifferentiated cells, their reduced cell clumping in culture, their increased shear stress resistance and ability to routinely secrete high levels of paclitaxel into the cultivation media, facilitating subsequent purification (Hirasuna et al. 1996).

### Plant cell cultivation using in situ product recovery

In situ product recovery (ISPR) has been utilised for the bioprocessing of diverse biomolecules (antioxidants, antitumor, alkaloids and antibiotics, among others) from a variety of culturable organisms including bacteria, yeast and plant cells (Phillips et al. 2013; Liu et al. 2021; Santoyo-Garcia et al. 2022). To achieve ISPR, different extractants or adsorbers are applied at different cultivation points, including liquid extractants (limonene or dodecane), solid extractants (charcoal, activated clay, sylopute, macro-porous resins and 3D-printed material) and even foam (Pyo et al. 2005; Brennan et al. 2012; Najmi et al. 2018; Kang and Kim 2021). A technique that has proven to be especially effective in the recovery of paclitaxel using *Taxus* cell suspension cultures cells is the application of the macro-porous resin, XAD-4, where the use of the resin at day 7 of cultivation increased paclitaxel production by up to 70%, reaching concentrations of 2.7 mg/L (Kwon et al. 1998). Other studies using solid adsorbents discovered that the use of sylopute (SiO_2_) as adsorbent material in the extraction step enhanced the paclitaxel yield by 30 to 45% compared to methods with no adsorbent treatment (Min and Kim 2022). In the context of microbial cells, cultures of *Aspergillus fumigatus* and *Alternaria tenuissima* have been immobilised using different entrapment carriers (gelatin, agar and Arabic gum), increasing paclitaxel yields by 1.3- and 1.8-fold, respectively, compared to free cultures (El-Sayed et al. 2019). In engineered yeast, the effectiveness of combining different macro-porous resins with different surface polarity increased the titre recovery of different taxanes reaching an eightfold increase using this ISPR approach (Santoyo-Garcia et al. 2023).

The application of combined resins during ISPR cultivation has not been studied in either cultured DDCs cells or VSCs. We therefore determined the potential utility of this ISPR approach for VSCs.

In addition to increasing target product recovery, the ISPR approach has also been shown to be effective in removing cell-waste compounds and reactive oxygen species (ROS) produced by the culturable organism (Ochoa-Villarreal et al. 2016; Santoyo-Garcia et al. 2022). This is significant, as some products/ROS can activate secondary undesired pathways, inhibit cell growth or divert the metabolic flux towards side products (Jiang et al. 2019).

While in our previous work (Santoyo-Garcia et al. 2023) we reported the effectiveness of the combination of resins HP-20, XAD7HP and HP-2MG in recovering taxanes from yeast cell factories, it is important to mention that our findings have not been directly applied to plant VSCs cells. The physical–chemical differences of these macro-porous commercial resins can be found in Santoyo-Garcia et al. (2023). In addition, the main physical–chemical properties are described in Table 1.

**Table 1:** Physical–chemical characteristics of the three resins used in the ISPR cultivations

Resin	Particle size (μm); density	Pore size (Å)	Composition	Surface area (m^2^/g)	Reference
HP-20	400 to 1000; 1.01	290	Polystyrene and divinylbenzene	720	(Phillips et al. 2013)
XAD7HP	560 to 710; 1.05	90	Acrylic ester	450	(Phillips et al. 2013)
HP-2MG	500 to 600; 1.09	170	Methacrylic ester copolymer	500	(Soto et al. 2012)

**Fig. 1:**
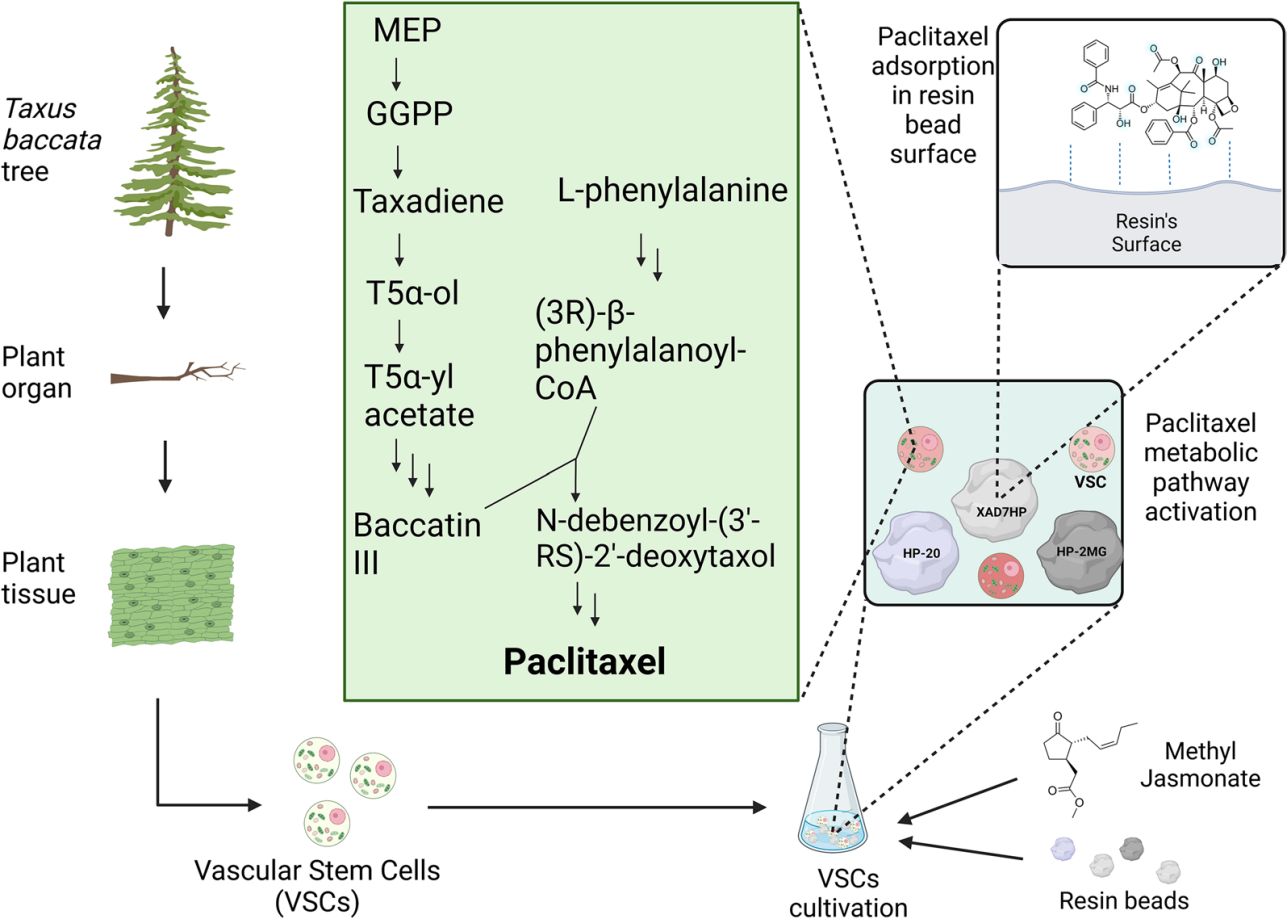
In situ product recovery (ISPR) of paclitaxel from *T. baccata* vascular stem cells (VSCs) using a combination of resins. Paclitaxel production occurred only after the addition of the elicitor methyl jasmonate (Me-JA). The addition of the “elicitor” Me-JA and the resins was undertaken simultaneously to sequester the taxanes produced by the VSCs

Here, novel ISPR cultivation approaches were applied for the first time to VSCs of *T. baccata* to potentially improve paclitaxel production and recovery, as described in Fig. 1. Our primary objective was to increase taxanes yields, not only in yeasts, as previously reported (Santoyo-Garcia et al. 2023), but also in various other productive organisms, such as plants cells. This process aims to elucidate the resin's influence on taxanes recovery, based on its adsorption characteristics for the particular metabolites generated by the plant cell line.

After determining the best resin combination and concentration in the batch approach, semi-continuous cultivations combined with optimised ISPR were established, which significantly increased the yield of paclitaxel.

## Results and discussion

### Cell growth and taxane production both pre-elicitation and post-elicitation utilising distinct macro-porous resins

**Fig. 2:**
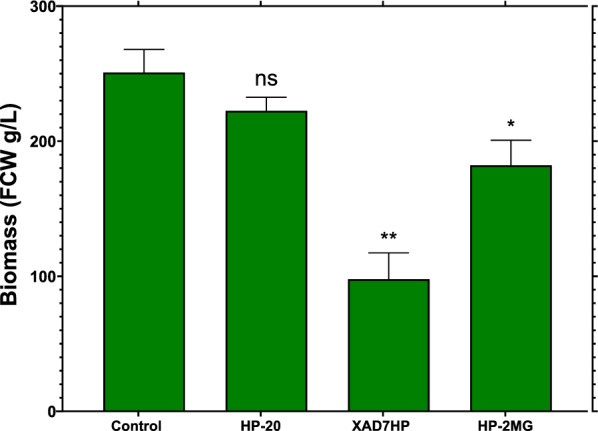
Pre-elicitation cell growth in the presence of different single adsorbent resin beads. Fresh cell weight (FCW) after 14 days of cultivation at 25 °C and 100 rpm in a dark environment for each given resin is shown. All treatments used 3% w/v of single resin beads, where error bars represent S.D. (*n* = 2). Statistical analysis was made using Dunnett’s method (ordinary one-way ANOVA) where all bars were compared against control where *p* < 0.001 ***, *p* < 0.02 **, *p* < 0.03 * and *p* > 0.1 ns

*Taxus baccata* VSCs were set in different resin bead treatments at different growth stages to identify the optimal approach for both cellular growth and taxane production. Thus, VSCs were placed in contact with single resin beads, preceding elicitation, for 14 days to determine the effect of each resin on the growth of VSCs compared to those not exposed to the given resin. The resulting data were utilised to select the optimum resin combination with respect to the final biomass accumulation and visible cell stress markers, e.g. cell “browning” (Onrubia et al. 2013). These results are summarised in Fig. 2.

After 14 days of cultivation, it was observed that the application of all adsorbent resins to VSCs had an inhibitory growth effect as determined by final fresh weight. The control sample, cultured in the absence of resin, generated the highest biomass accumulation at 250 ± 12 g/L. Further, the associated VSCs exhibited no visible stress markers. VSCs cultured in the presence of HP-20 had no significant difference from control (*p* = 0.332) which had 222 ± 7 g/L. In addition, biomass accumulation in treatment using HP-2MG was statistically different from the control with 182 ± 13 (*p* = 0.03). Interestingly, the resin that showed the lowest biomass accumulation and higher significant difference (p = 0.002) was XAD7HP resin with 98 ± 13 g/L of fresh cell weight (Fig. 2). However, in the presence of this resin VSCs exhibited the most healthy appearance, with no “cell browning”, indicating low stress levels (Additional file 1: Fig. S4). This discrepancy among resins could result from the differential generation of shear stress (Shi et al. 2003). Another plausible explanation is that XAD7HP resin may have a higher affinity for adsorbing the media nutrients, which could subsequently exert an inhibitory effect on cell growth (Additional file 1: Fig. S4).

**Fig. 3:**
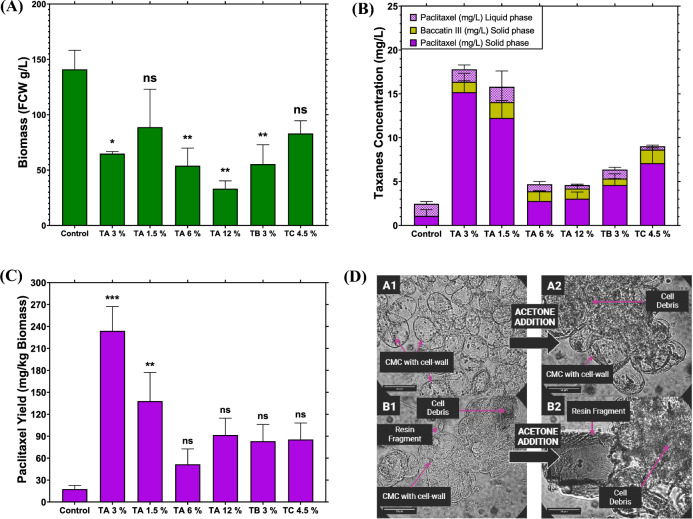
Cell biomass accumulation and taxane accumulation using different adsorbent resin combinations from Table 2. **A** Biomass measured as fresh cell weight (FCW) of the VSCs after 14 days of cultivation. **B** Concentration of paclitaxel and baccatin III in the solid phase (cells and resins) and the liquid phase. **C** Total paclitaxel recovered against the final biomass in each treatment. Bars represent the mean of the experiments (*n* = 2) and the error bars of S.D. Statistical analysis was undertaken using Dunnett’s method where all bars were compared against the control, where *p* < 0.001 ***, *p* < 002 **, *p* < 0.03 * and *p* > 0.1 ns. **D** Microscopic images of VSCs in the control (A1 and A2) and treatment A (B1 and B2) before and after adding the acetone solvent at 99%

Next, we determined the impact of combinations of these resins on in situ cultivation of VSCs at different resin concentrations and configurations to test the impact on both cell growth and taxane production, as the combination of these three resins has shown favourable results for purification of taxanes from recombinant yeast expressing some of the known paclitaxel pathway enzymes (Santoyo-Garcia et al. 2023). Treatments consisting of three combinations of resins were employed (Table 1). Treatment A had a majority of the polar resin HP-2MG. This specific combination was selected as it has been shown to be effective in the recovery of taxanes from yeast cell factories (Santoyo-Garcia et al. 2023). As demonstrated in previous studies, the total concentration of the resins in the culturable cells is a critical variable for cell growth (Yin et al. 2005; Santoyo-Garcia et al. 2022). For that reason, treatment A configuration (0.5, 1 and 1.5% w/v of HP-20, XAD7 and HP-2MG, respectively) of resins at different total concentrations (1.5, 3 and 6% *w*/*v*) was utilised to test cell growth and taxanes production in VSCs. Treatment B had a majority of the resin XAD7HP, because our data have demonstrated that in pre-elicitation experiments this resin results in a lower biomass accumulation but significantly improved cell health maintenance (Fig. 2 and Additional file 1: Fig. S4). Finally, treatment C was derived from treatment A with the difference of increasing the polar resin concentration HP-2MG (up with a total resin concentration of 4.5% *w*/*v*) to test if utilising more polar resins would increase the recovery of paclitaxel. All treatments were run simultaneously in 6-well plates along with a control treatment with no adsorbent resins in the fermentation. These treatment details are summarised in Table 2. After the cultivation period, a measurement of the biomass fresh weight and taxane recovery was performed as shown in Fig. 3.

**Table 2:** Resin combination treatments. Details of each resin treatment is as indicated

Treatment	HP-20 resin (% *w*/*v*)	XAD7HP resin (% *w*/*v*)	HP-2MG resin (% *w*/*v*)	Total Resin in cultivation (% *w*/*v*)
A1	0.5	1	1.5	3
A2	0.25	0.5	0.75	1.5
A3	1	2	3	6
A4	2	4	6	12
B	0.5	1.5	1	3
C	0.5	1	3	4.5
Control	0	0	0	0

After “elicitation” using Me-JA, a potent endogenous plant immune activator, the biochemical pathway associated with paclitaxel biosynthesis is transcriptionally activated. This also results in a decrease in the overall cell growth rate, as cellular resources and directed towards immune responses rather than plant growth (Cusido et al. 2014). As shown in Fig. 3A, the biomass accumulation in all the treatments was reduced relative to that in the absence of Me-JA treatment (Fig. 2). Figure 3A also shows that combining Me-JA elicitation and the application of resins results in lower biomass yields of VSCs following the cultivation period. As previously reported (Shi et al. 2003; Ochoa-Villarreal et al. 2016; Santoyo-Garcia et al. 2022), the quantity of the resin in an in situ cultivation will affect the viability of cultured cells. This is reiterated in the biomass accumulation recorded for treatment A, where 3, 6 and 12% *w*/*v* resins were applied, resulting in a significant difference compared to control in the biomass yields at 65 ± 1 (*p* = 0.015), 54 ± 11 (*p* = 0.008) and 33 ± 5 g/L (*p* = 0.002), respectively. In contrast, the control treatment, where no resin was administered, had the highest biomass yield following Me-JA treatment at 141 ± 12 g/L of fresh weight (Fig. 3A).

Our data indicated that the treatment resulting in the highest purification levels of taxanes was treatment A, with 1.5 and 3% *w*/*v* with 16 and 18 mg/L, respectively, which indicates an average 8.5-fold increase in comparison to the control, in the absence of added resin. Interestingly, taxane partition was different in the treatments using resins compared to the control treatment, as almost half of the taxanes produced in the control were in the solid phase (only biomass), while the rest of the taxanes were found in the liquid phase (Fig. 3B). In contrast, the taxane partition in the resin treatments had an average of 80 to 90% of the taxanes in the solid phase (biomass and resins); these results are in agreement with previous studies where paclitaxel was extracted using in situ cultivations with XAD resins and 60% was found in the adsorber (Kwon et al. 1998). The above data could result from the resins sequestering taxanes secreted from VSCs into the media, decreasing the concentration of free taxanes in the media that might (1) either be directly toxic to VSCs or (2) negatively feedback-regulate the production of taxanes.

Next, the total paclitaxel obtained in the liquid and solid phase was summed and divided by the final biomass of each treatment to obtain the final paclitaxel yield in (mg) per kg of biomass, as shown in Fig. 3C. The fermentation that produced the best yield was treatment A at 3% *w*/*v* with 234 ± 23 mg/kg compared to the 17.5 ± 3 mg/kg obtained in the control treatment (*p* < 0.001), which represents an improvement of 13-fold. This result was compared with previous studies that utilised cultured *Taxus* cells to obtain paclitaxel: this method was found to be 3.7-fold higher relative to that obtained with *Taxus cuspidata* cell cultures (Wang et al. 2016) and 2.3-fold higher relative to *Taxus cuspidata* cambial meristematic cells (CMCs) (Howat et al. 2014). Thus, our approach enables increased paclitaxel yields when compared to previous studies. The higher titres of paclitaxel could be explained by the continuous removal of paclitaxel from the cells, and also potentially by the disruption of the cells during the cultivation and at the extraction/desorption stage as the treatments that used resins were under constant mechanic stress with the cells provoking an integration of the bioprocess in the extraction step (Zainuddin et al. 2021). This effect is shown in Fig. 3D, where cell lysis can be observed during the cultivation process in the treatment with resins (Fig. 3D-B1). In contrast, in the control cultivation, where cells presented a visibly healthy morphology, the cell wall was intact (Fig. 3D-A1). However, in the control, after adding the pure acetone solvent for extraction, not all the VSCs lysed compared to the resin treatment, where lysis was complete. Thus, there appeared to be a synergistic impact of cell disruption between the acetone solvent and mechanic stress resulting from the resin treatment (Fig. 3D-A2 and B2).

### Accumulation of reactive oxygen species during fermentations

**Fig. 4:**
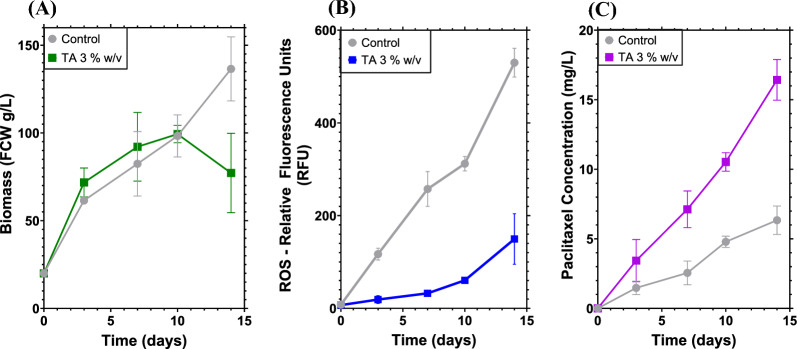
ISPR cultivation kinetics of VSCs after elicitation. **A** Biomass in fresh cell weight (FCW) (g/L). **B** Accumulated reactive oxygen species (ROS) in relative fluorescence units. **C** Paclitaxel concentrations in the ISPR fermentation after elicitation with Methyl Jasmonate (Me-JA). Experiments were undertaken in parallel with Treatment A at 3% *w*/*v* resins and the control with no resins. All runs were performed under the same conditions: 25 °C, 100 rpm, under darkness and identical media compositions. Points represent the mean value, where error bars are S.D. (*n* = 3)

We also measured biomass, ROS and paclitaxel production throughout the fermentation following treatment A relative to the control (Fig. 4).

**Fig. 5:**
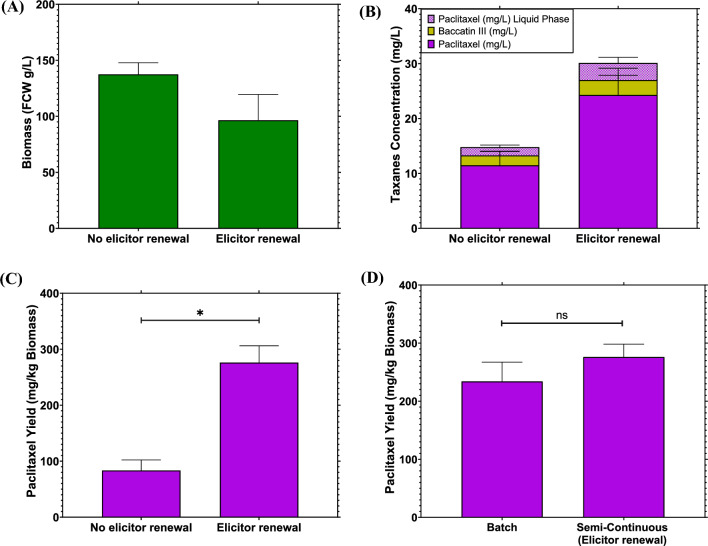
Semi-continuous experiments to test biomass and taxane production. **A** Biomass in fresh cell weight (FCW) accumulated at the end of each cultivation. **B** Total taxane concentrations in the cultivation phases (solid and liquid). **C** Paclitaxel yields from biomass of VSCs. **D** Comparison of batch and semi-continuous (with elicitor renewal) paclitaxel yields. The semi-continuous runs lasted 5 weeks in total including three media renewals, with conditions of 25 °C, 100 rpm in a dark environment and the same media concentration as batch cultivations. The resin treatment used was treatment A at 3% *w*/*v* consisting of 0.5, 1 and 1.5% *w*/*v* of HP-20, XAD7HP and HP-2MG, respectively. Bars represent the mean value, while the error bars represent S.D. (*n* = 3). Paired *t*-test was used for statistical analysis in (**C**) (*t* = 8.64) and **D** (*t* = 1.075) where *p* < 0.001 ***, *p* < 002 **, *p* < 0.03 * and *p* > 0.1 ns

After adding the elicitor, Me-JA, at a final concentration of 100 μM, metabolic reprogramming occurred in VSCs switching biochemical flux from growth to immune responses (Lee et al. 2010). This effect could be appreciated in Fig. 4A, where the biomass accumulation in every sample point was lower than cell growth in Fig. 2. The final biomass values in Fig. 4A were similar to the results obtained in Fig. 3A where the control was 136 ± 13 g/L compared to the 77 ± 16 g/L of treatment A at 3% w/v. As previously explained, the higher titre of biomass in the control cultivation (Fig. 4A) could be due to the reduced mechanical stress and increased availability of nutrients due to a lack of absorbing resins in the media. Moreover, as the concentration of resins increases, the cell growth diminishes. This trend is evident in Fig. 3A, where in treatment A, the lower resin concentration exhibited a more substantial increase in biomass. This could be attributed to the interplay of shear stress and nutrient adsorption from the resins, influencing the biomass concentration in this particular resin combination. Interestingly, both the intracellular and extracellular ROS levels were higher in the control treatments (Fig. 4B). Further, the data indicate that the applied resins were adsorbing the ROS quantified: oxygen ions (O^−^) and hydroxide peroxide (H_2_O_2_) (Shi et al. 2003). It has been reported that the accumulation of ROS in *Taxus* cultures increases permeability and lipid peroxidation of the membrane and that the interaction of ROS with cultured cells might promote the phenylalanine ammonia-lyase (PAL) pathway, which could increase paclitaxel production (Yin et al. 2005). However, in the presence of resins, a constant Me-JA concentration (100 μM) in combination with a low ROS level, paclitaxel accumulation was increased to 16.4 ± 1 mg/L compared to 6.3 ± 1 mg/L obtained by the control treatment, with a 2.6-fold increase in ROS (Fig. 5B and C). Previous studies have stated that a higher ROS concentration could improve paclitaxel production as ROS could act as an elicitor activating the *Taxus* defence mechanisms (Han and Yuan 2004; Yin et al. 2005). However, in this work, the ROS concentration in the media appeared to be diminished by a solid absorbent, which favoured paclitaxel production as shown in Fig. 5B and C. This increase in paclitaxel could be explained as the cells appeared visibly healthier and taxane production by VSCs was enhanced by having less ROS in the media, a low shear stress provoked by the resins and a constant concentration of the Me-JA in the cultivation.

The ROS effect could also explain the results presented in Fig. 4C, where the paclitaxel concentrations throughout the control cultivations was lower than treatment A samples at almost all time points. At the end of the cultivation, treatment A had 16.4 ± 1 mg/L of paclitaxel, which was 2.6-fold higher than the 6.3 ± 0.7 mg/L of paclitaxel in the control.

### Semi-continuous paclitaxel production

Recently, there has been a shift towards semi-continuous and continuous bioprocesses (Wilk et al. 2019; Teworte et al. 2022). In an attempt to further improve paclitaxel synthesis, a semi-continuous experiment was performed. The cycles for media renewal were established in two ways: by maintaining a constant Me-JA concentration and in addition, undertaking renewal by the introduction of fresh media (Fig. 5).

The use of Me-JA in the renewal-media cycles had an important impact on biomass accumulation, as well as on the yield of paclitaxel (Fig. 5A–C). The biomass accumulation in the treatment with no elicitor renewal in the cycles was 137 ± 8 g/L and 96 ± 18 g/L of VSCs with the elicitor renewal cycles, which indicates a 1.75-fold increase of biomass in the treatment with the diluted elicitor (Fig. 5A). This result agrees with previous studies demonstrating how Me-JA concentration has an important effect on biomass accumulation and paclitaxel yield in the Taxus cultures (Wang et al. 2016). Taxane production in VSCs was higher in the treatments that maintained the Me-JA concentration in the media renewal cycles. It is shown in Fig. 5B that the treatments that maintained the elicitor concentration at 100 μM had a total taxane concentration of 30 ± 6 mg/L. Meanwhile, treatments that did not have elicitor in the media renewal cycle presented a total taxane concentration of 15 ± 2.5 mg/L, representing a twofold decrease in total taxane concentration. These results highlight the importance of maintaining a consistent concentration of the elicitor throughout the process. In the absence of elicitor renewal, there is a decline in paclitaxel concentration with each cycle, as depicted in Fig. 5C. It is worth mentioning that as seen in the batch cultivations, the total taxanes in the solid phase (biomass and resins) represented around 85% of the total taxanes recovered in the experiments. This result agrees with previous studies where the majority of the target natural product was in extractant rather than the media or liquid phase of the fermentations (Kwon et al. 1998; Kim and Kim 2019). Further, the precursor baccatin III was only detected in the solid phase of the cultivation.

When total paclitaxel is divided by the total biomass obtained at the end of the cultivation, it can be seen that the treatment with elicitor renewal had 276 ± 24 mg/kg of paclitaxel, which was 3.3-fold higher than the 83 ± 15 mg/kg of paclitaxel obtained in the treatment with no elicitor renewal making it statistical different (*p* = 0.01). This difference between yields was likely due to the lower biomass in the elicitor renewal treatments and higher paclitaxel titres compared to the treatments with no elicitor renewal. The highest paclitaxel titre obtained in this study was 2.7-fold higher compared to the 102 mg/kg of paclitaxel obtained from VSCs of *Taxus cuspidata* (Howat et al. 2014). Finally, the result of 276 mg/kg in this study was 1.2-fold higher than the highest paclitaxel reported of 228.6 mg/kg using *Taxus baccata* suspension cells (Yukimune et al. 1996) and similar to the 264 mg/kg of paclitaxel at day 45 from VSCs of *Taxus cuspidata* (Ochoa-Villarreal et al. 2016).

Semi-continuous experiments were performed by leaving the resins in the plate wells and removing only cells and media that have been demonstrated to increase taxane titres in other microbial organisms compared to batch cultivations. Furthermore, the utilisation of ISPR resulted in the retention of over 99% of the produced taxanes within the solid phase, encompassing both resins and biomass (Santoyo-Garcia et al. 2023). Therefore, the paclitaxel concentration in the liquid phase was found to be negligible and was discarded. Nevertheless, the yields of paclitaxel in Fig. 5D showed that the difference between batch and semi-continuous titres was not statistically different. It is important to mention that the observed differences between semi-continuous and batch cultivations were not limited to paclitaxel yield; they also encompassed the appearance of VSCs. Stressed cells exhibited more pronounced color changes, which were visible in the extracts and led to a greater number of chromatogram peaks during HPLC analysis (Additional file 1: Figs. S3 and S5). In semi-continuous cultivations, VSCs turned a reddish colour and the resins became intensely pigmented, whereas in batch cultivations, VSCs appeared healthy and non-stressed, having a creamy colour with reddish resins (Additional file 1: Fig. S5). This could mean that the semi-continuous cultivation affected VSC metabolic pathways in other than those connected with NP synthesis, which did not translate into a significant increase in paclitaxel accumulation.

## Conclusions

The use of different solid macro-porous resins in the ISPR cultivations of VSCs has been shown to be effective in paclitaxel recovery. The optimum resin combination resulted in 0.5%, 1% and 1.5% *w*/*v* of HP-20, XAD-7HP and HP-2MG, respectively, for VSC cultivations, adding up a total of 3% *w*/*v* of the combined resins in the media. This resins treatment resulted in lower biomass accumulation compared to the control; nonetheless, it also resulted in 234 ± 23 and 276 ± 24 mg/kg of paclitaxel in batch and semi-continuous cultivations, respectively, representing a 13-fold and 16-fold improvement of paclitaxel yield compared to control without the use of extractive resins. These paclitaxel titres were the highest to be reported from plant cultivation tissue using VSCs in cultivations of less than 1 month. The increase in paclitaxel titres could be explained by the efficacy of the ISPR treatment in removing cell waste at the end of the cultivation, which displayed fourfold less ROS than the control cultivations. This important result was contrary to previous studies claiming that the presence of a high concentration of ROS in plant cells could mean more paclitaxel production. To enhance the reliability and accuracy of captured metabolites, future work should also be focus on adopting diverse purification methods, including techniques such as UHPLC-MS/MS (Gai et al. 2020) or LC–MS/MS (Wang et al. 2011; Dalmaris et al. 2019, 2020) for the identification and quantification of additional taxanes within the mixture. Furthermore, the exploration of alternative elicitors beyond Me-JA, such as salicylic acid, could also be contemplated for further study.

Finally, microscopic analysis showed a cell lysis effect of combining the resin's mechanical stress with acetone in the extraction step resulted in enhanced cellular disruption of VSCs, which may have promoted paclitaxel recovery. In our previous research (Santoyo-Garcia et al. 2022), we demonstrated that a similar ISPR approach was scalable to bench top bioreactors using *S. cerevisiae*. Therefore, conducting scale-up studies using VSCs would aid in determining the industrial application and economic viability of this integrated bioprocess.

## Materials and methods

### *Taxus baccata* vascular stem cells and media

The *Taxus baccata* vascular stem cells (VSCs) were cultivated according to a methodology adapted from (Lee et al. 2010). Briefly described, VSCs were isolated from wild-type *Taxus baccata* twigs. Twigs were disinfected by using different steps described by Lee et al. 2010. After disinfection, cambium, phloem, cortex and epidermal tissue were peeled away from the xylem. All the cells were cultured in B5 medium excluding (NH_4_)2SO_4_ with 1 mg/L picloram, 30 g/L sucrose and 4 g/L gelrite (Gamborg et al. 1968). After 30 days of cultivation cambium cells were separated from the rest of the cells as it forms a clear split between phloem, cortex and epidermis cells. After isolating the cambium cells, the cells were cultivated onto different Petri dishes containing B5 medium excluding (NH_4_)2SO_4_ with 1 mg/L picloram, 10 g/L sucrose and 4 g/L gelrite. VSCs were sub-cultured onto the fresh medium every 2 weeks by using 3 g of fresh cell weight (FCW) inoculum into 125 mL Erlenmeyer flasks containing 25 mL of B5 medium (Lee et al. 2010).

The B5 cultivation media composition is shown in Additional file 1: Table S2 (Lee et al. 2010).

### In situ vascular stem cells batch cultivation pre-elicitation

To test the effect of the single resins on the growth of the *T. baccata* VSC suspension culture, independent cell cultures were prepared containing a final concentration of 3% w/v of the resins HP-20 (non-polar), XAD7HP (amphipathic) and HP-2MG (polar), respectively. These resins have been used in previous studies to recover oxygenated taxanes (Santoyo-Garcia et al. 2023). The VSC suspension cultivation was previously cultured in 250 mL shake flasks using 20% working volume. Subcultures were made every 2 weeks to maintain cells in optimal conditions. For every treatment, a 5 mL sample from the standard cell solution was transferred into the well of a sterile 6-well cultivation plate (Corning, USA) at a cell density of 0.1 mL of cells/mL. Subsequently, the previously weighted and autoclaved resins were added to each treatment to maintain sterility. The in situ VSC cultures were incubated at 25 °C and 100 rpm in the dark for 14 days in a platform shaker (Innova 2000, New Brunswick Scientific, USA). The treatments (using single resins at 3% *w*/*v*) were performed in duplicate. At the end of the cultivation period, the biomass fresh cell weight (FCW) was measured and the cell's colour and morphology were analysed to optimise the resin combinations.

### In situ vascular stem cells batch cultivation post-elicitation

VSCs were cultured using the same conditions as stated in Sect. “In situ vascular stem cells batch cultivation pre-elicitation” with some modifications where after transferring the 5 mL of working volume into the 6-well plate, no resins were added and 7 days were set before making the elicitation for the cells to adapt to the new conditions. Subsequently, the cells were elicited by adding previously sterilised and pure Methyl Jasmonate (Me-JA) (Merck, Germany) using a syringe filter (0.22 μm) to reach a final concentration of 100 μM in each treatment. Simultaneously, previously weighted and autoclaved resins (HP-20, XAD7HP and HP-2MG) were added according to the treatment for incubation for 14 days to reach total cultivation of 21 days.

**Fig. 6:**
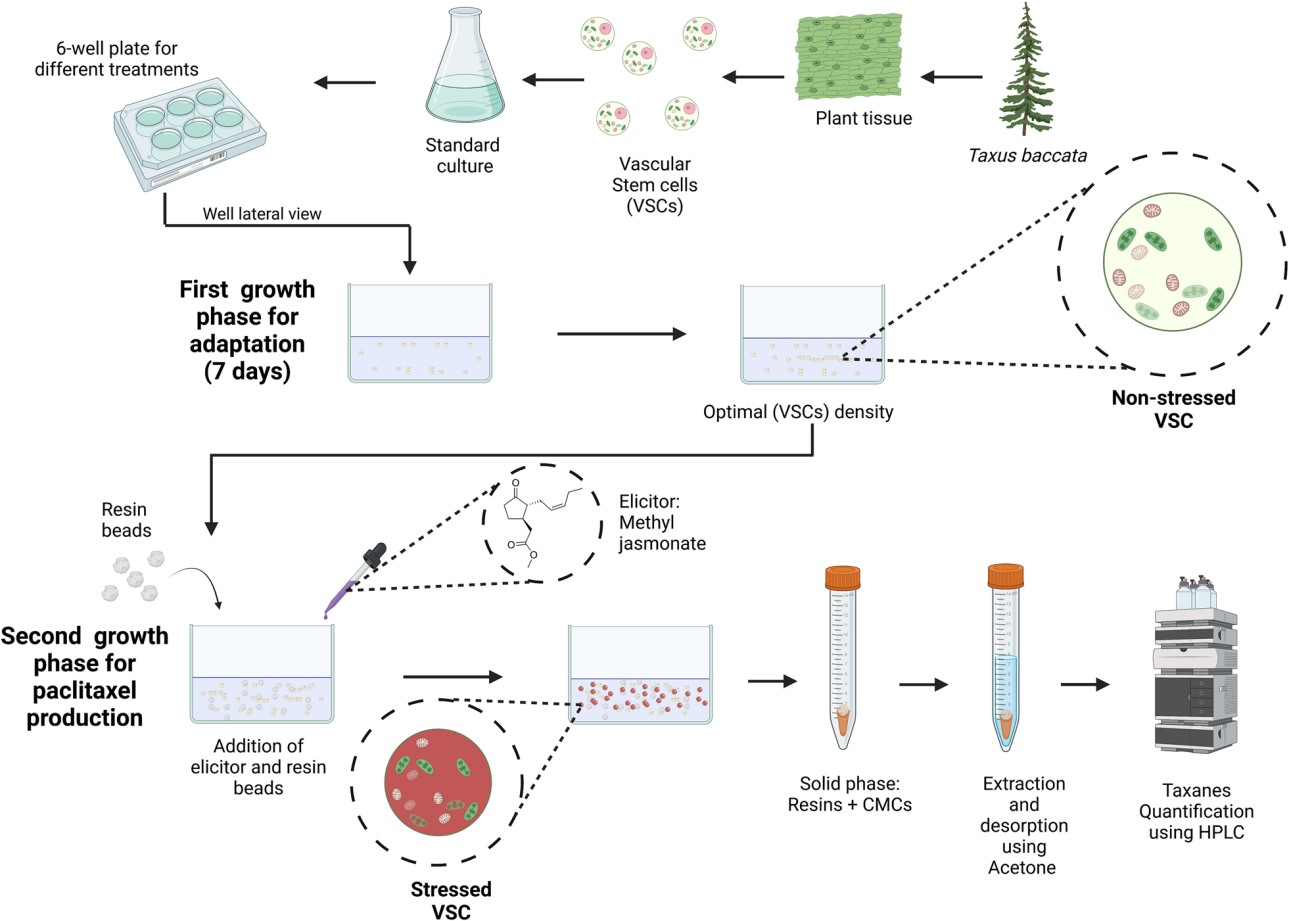
VSCs *Taxus baccata* growth using the ISPR method. Elicitor and resins were added at the same cultivation time after 7 days of VSCs adaptation. Elicitor used was Methyl Jasmonate and the resins used were HP-20, XAD7HP and HP-2MG

The standard cell solution (obtained from previous subcultures of VSCs that were around the 7th to the 10th day of cultivation) and treatments were combined and incubated under 100 rpm at 25 °C in darkness in a platform shaker (Innova 2000, New Brunswick Scientific, USA). The control treatments followed the same methodology without the addition of any resin at any point during the cultivation. For kinetics measurements, sacrifice sampling was used in the 6-well plates. Biomass was measured by weighing the VSCs in pre-weighted falcon tubes to obtain the fresh weight at the end of cultivation. Microscope images were taken using an oil immersion brightfield microscope equipped with a 50 × Leica NPLAN objective lens (Leica Microsystems, Germany) using an Andor Zyla sCMOS camera (Oxford Instruments, UK). The methodology used in this study is described in Fig. 6.

### Semi-continuous VSCs cultivation

VSCs were treated the same in semi-continuous cultivations as in batch cultivations until reaching the elicitation point. After the elicitation, three renewal-media cycles were performed in the treatments. Each cycle comprised incubating the VSC suspension cultivation for 7 days, followed by the removal of 70% of the cultivation volume by careful pipetting that only involved media and cells, leaving the resins in each cultivation well. After the media and cell removal, the same volume of fresh media was added to each treatment. Two types of media renewals were used in the cycles, one being only the growth fresh media and the other with the same fresh media but with the Me-JA maintained at 100 μM. After the cycles, the treatments were further incubated for 14 days to end the cultivation in a total of 5 weeks.

### Extraction and desorption of paclitaxel and other taxanes

After the cultivation period of the treatments, the content of each well was transferred to a 15 mL falcon tube. The liquid phase was separated after waiting 10 min of not moving the tubes to let the solid phase (cells + resins) sediment in the bottom. After separating the phases, the liquid phase was centrifuged at 21,500 rpm for 10 min, followed by obtaining an aliquot of 100 μL from the liquid phase and transferring it for HPLC analysis. The solid phase was weighted to determine the fresh weight, while 99.9% acetone (Fisher Scientific, UK) was added to make the solid–liquid extraction from the cells and resins for 12 h in a shaking cabinet at 250 rpm at 25 °C (Innova 42, Eppendorf, UK). After the extraction period, the tubes were centrifuged at 21,500 rpm for 10 min, and then 100 μL of the supernatant was transferred for further HPLC analysis.

### Reactive oxygen species detection

ROS quantification was made at different cultivation points for treatment with resins and control with no extraction aid. The intracellular fluorescence method (Abhishek and Elah 2021) was used with some modifications. At the specific cultivation times of 0, 3, 7, 10 and 14 days, a homogeneous aliquot (with no resins) of 100 μL was separated from the rest of the cultivation media. Then, 4 μL of 2ʹ,7ʹ-dichlorodihydrofluorescein diacetate (H_2_-DCFDA) solution (Invitrogen, USA) was added from a stock solution (5 mg/mL) and incubated at 30 °C for one hour. After that, centrifugation was made at 4500 rpm for 5 min to keep only the pellet that was resuspended and washed two times with 500 μL of PBS 1X (Merck, Germany) to finally add 200 μL of PBS. This solution was transferred to a dark 96-well plate (Greiner Bio-one, Germany) for fluorescence reading by exciting at 484 ± 10 nm and emission at 518 ± 10 nm wavelengths in a microplate reader (FLUOstar Omega, BMG Labtech).

### Analytical methods for taxane detection and quantification

For taxane detection and quantification, a semi-preparative HPLC Agilent 1100/1200 (Agilent Technologies, USA) was used. The equipment had five modules: Degasser, Quaternary pump, Auto-sampler, Column heater and Diode Array Detector (DAD). The packed column used was a C_18_ Discovery (Merck, Germany, 250 × 4.6 mm and 5 μm particle size). 5 μL of the extract samples was injected using an auto-sampler at 1 mL/min with a gradient method of the mobile phase that went from 95% of solvent A (pure water) until reaching 40% of the same solvent and 60% of solvent B (Acetonitrile 99%) (Fisher Scientific, UK) the first 4 min and keeping that isocratic concentration until the end of the run at 12.5 min. The column compartment was set up to 60 °C. The detection of paclitaxel and baccatin III was performed at a wavelength of 270 nm. Calibration curves of paclitaxel and baccatin III were performed using standard pure compounds (Merck, Germany) (Additional file 1: Fig. S1). HPLC chromatograms for taxane (baccatin III and paclitaxel) retention times and method regarding the HPLC runs are detailed in Additional file 1: Figs. S1 and S2, as well as Additional file 1: Table S1.

### Statistical analysis

Statistical analyses were performed using GraphPad Prism 8.0 software. One-way analysis of variance (ANOVA) was used to determine whether resin beads combination treatment yielded a significant impact on paclitaxel titre to select the treatment for semi-continuous cultivation. A Dunnett’s multiple comparison test was used to compare each treatment to the control (with no resins). One-way ANOVA was also used to determine whether the selected extraction solvent influenced taxadiene titres recovery. Paired t-test was subsequently employed to compare batch and semi-continuous treatments. The null hypothesis considered that there was no significant difference between the treatments; hence, if *p* ≤ 0.05, the null hypothesis was rejected.

### Supplementary Information

**Additional file 1: Figure S1. **Calibration curves for Paclitaxel (**A**) and Baccatin III (**B**). HPLC chromatogram (**C**) for the standard solutions where paclitaxel showed a retention time of 9.6 min and Baccatin III of 7.95 min. **Figure S2.** HPLC Chromatogram for batch cultivation. Paclitaxel showed a retention time of 9.61 min and Baccatin III of 8.06 min. **Figure S3.** HPLC Chromatogram for semi-continuous cultivation. Paclitaxel showed a retention time of 9.7 min and Baccatin III of 7.99 min. **Figure S4.** In situ CMCs cultivations using single resins. **A** Was performed using HP-20, **B** with HP-2MG and **C** with XAD7HP. All cultivations were made using the same conditions (section 2.2) and resin concentrations (3% *w*/*v*). **Figure S5.** CMCs (**A** and **B**) and acetone extracts (**C** and **D**) appearances in control, batch and semi-continuous cultivations. **Table S1.** HPLC method description. **Table S2.** *Taxus baccata* B5 media composition.

## Data Availability

The datasets used and/or analysed during the current study are available from the corresponding author upon reasonable request.

## Abbreviations

CMC(s): Cambial meristematic cell(s)
VSC(s): Vascular stem cell(s)
ROS: Reactive oxygen species
FCW: Fresh cell weight
PAL: Phenylalanine ammonia-lyase
ANOVA: One-way analysis of variance
DAD: Diode array detector
HPLC: High-performance liquid chromatography
Me-JA: Methyl jasmonate
ISPR: In situ product recovery
S.D.: Standard deviation
