# Supplementary material for: Increased paclitaxel recovery from Taxus baccata vascular stem cells using novel in situ product recovery approaches
Source: Bioresour Bioprocess. 2023 Sep 29;10(1):68. doi: 10.1186/s40643-023-00687-8 (PMC10991628; doi:10.1186/s40643-023-00687-8)
Supplement: Supplementary file 1 — Additional file 1: Figure S1. Calibration curves for Paclitaxel (A) and Baccatin III (B). HPLC chromatogram (C) for the standard solutions where paclitaxel showed a retention time of 9.6 min and Baccatin III of 7.95 min. Figure S2. HPLC Chromatogram for batch cultivation. Paclitaxel showed a retention time of 9.61 min and Baccatin III of 8.06 min. Figure S3. HPLC Chromatogram for semi-continuous cultivation. Paclitaxel showed a retention time of 9.7 min and Baccatin III of 7.99 min. Figure S4. In situ CMCs cultivations using single resins. A Was performed using HP-20, B with HP-2MG and C with XAD7HP. All cultivations were made using the same conditions (section 2.2) and resin concentrations (3% w/v). Figure S5. CMCs (A and B) and acetone extracts (C and D) appearances in control, batch and semi-continuous cultivations. Table S1. HPLC method description. Table S2. Taxus baccata B5 media composition. [file 40643_2023_687_MOESM1_ESM.docx]

**Additional file**

**Increased paclitaxel recovery from *Taxus baccata* vascular stem cells using novel *in situ* product recovery approaches**

Jorge H. Santoyo-Garcia^a,b,*^, Marissa Valdivia-Cabrera^c^, Marisol Ochoa-Villarreal^c^, Samuel Casasola-Zamora^d^, Magdalena Ripoll^f,g^, Ainoa Escrich^h^, Elisabeth Moyano^h^, Lorena Betancor^f^, Karen J. Halliday^c^, Gary J. Loake^c,d^, Leonardo Rios-Solis^a,b,e,i,*^

^a^*Institute for Bioengineering, School of Engineering, University of Edinburgh, Kings Buildings, Edinburgh EH9 3FB, United Kingdom*

*^b^Centre for Engineering Biology, University of Edinburgh, Kings Buildings, Edinburgh EH9 3BF, United Kingdom*

*^c^Institute of Molecular Plant Sciences, School of Biological Sciences, University of Edinburgh, King's Buildings, Edinburgh EH9 3BF, United Kingdom*

*^d^Green Bioactives Ltd, Roslin Innovation Centre, Easter Bush Campus, Midlothian, EH*

*^e^School of Natural and Environmental Sciences, Molecular Biology and Biotechnology Division, Newcastle University, Newcastle upon Tyne NE1 7RU, United Kingdom*

*^f^Laboratorio de Biotecnología, Universidad ORT Uruguay, Mercedes 1237, 11100 Montevideo, Uruguay*

*^g^ Graduate Program in Chemistry, Facultad de Química, Universidad de la República, Uruguay*

*^h^Department of Medicine and Life Sciences, Universitat Pompeu Fabra, Barcelona 08003, Spain*

*^i^Department of Biochemical Engineering, The Advanced Centre for Biochemical Engineering, University College London, Gower Street, London, WC1E 6BT, UK*

*^*^Corresponding authors: leo.rios@ucl.ac.uk and jorge.santoyogarcia@roslintech.com*

Figure S1. Calibration curves for Paclitaxel (A) and Baccatin III (B). HPLC chromatogram (C) for the standard solutions where paclitaxel showed a retention time of 9.6 min and Baccatin III of 7.95 min.


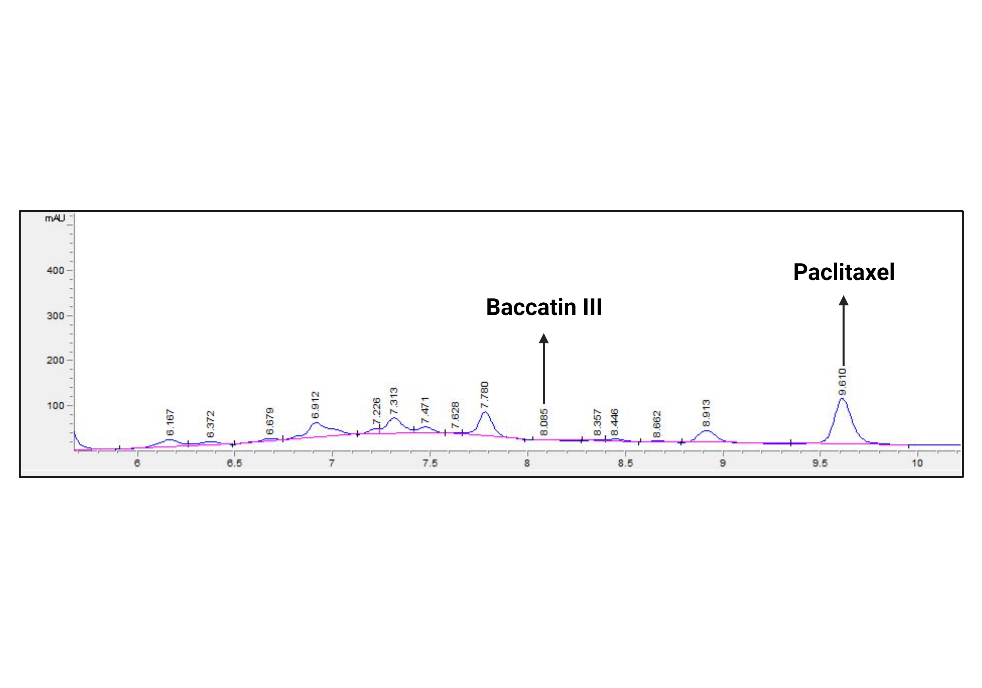


Figure S2. HPLC Chromatogram for batch cultivation. Paclitaxel showed a retention time of 9.61 min and Baccatin III of 8.06 min.


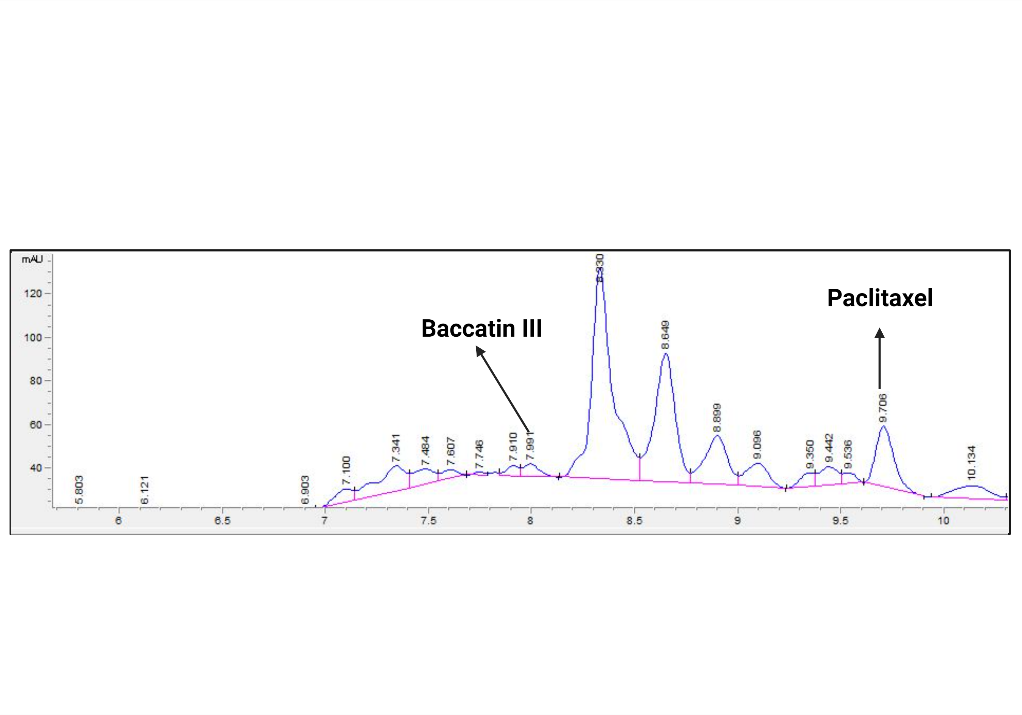


Figure S3. HPLC Chromatogram for semi-continuous cultivation. Paclitaxel showed a retention time of 9.7 min and Baccatin III of 7.99 min.

Figure S4. In situ CMCs cultivations using single resins. A) Was performed using HP-20, B) with HP-2MG and C) with XAD7HP. All cultivations were made using the same conditions (section 2.2) and resin concentrations (3% w/v).


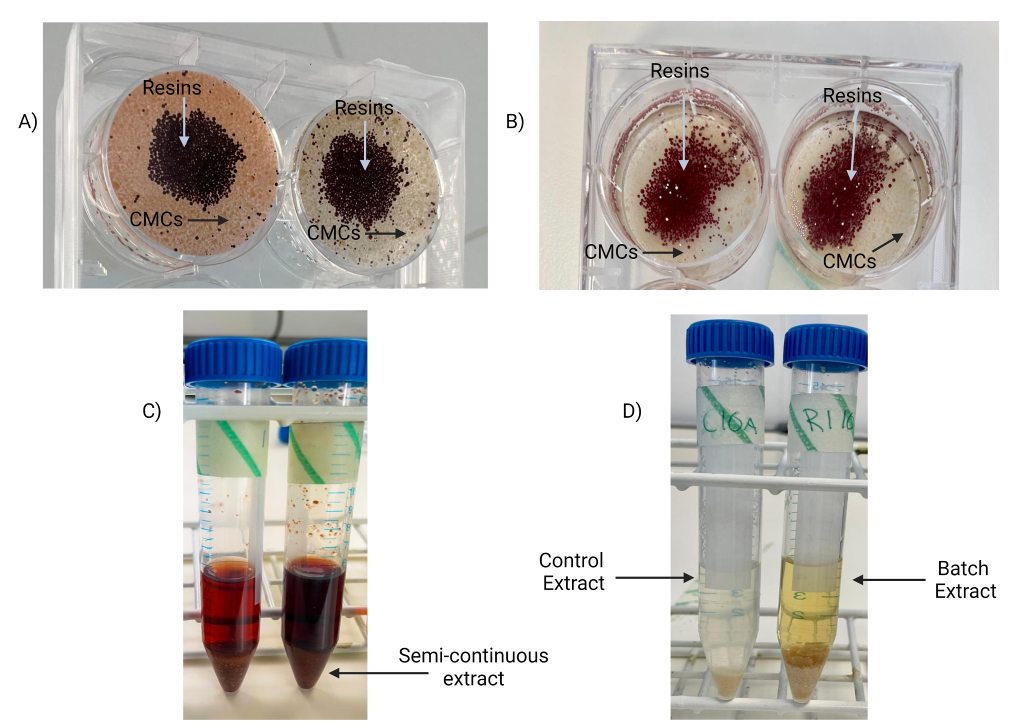


Figure S5. CMCs (A and B) and acetone extracts (C and D) appearances in control, batch and semi-continuous cultivations.

Table S1. HPLC method description.

|  | Run parameters | | |
| --- | --- | --- | --- |
| Time (min) | % Acetonitrile | % Water | Flow (ml/min) |
| 0 | 5 | 95 | 1 |
| 4 | 60 | 40 | 1 |
| 12 | 60 | 40 | 1 |
| 12.5 | 5 | 95 | 1 |
| 15.5 | 5 | 95 | 1 |

Table S2. *Taxus baccata* B5 media composition.

| Reagent | Concentration (mg/L) |
| --- | --- |
| Potassium nitrate | 2500 |
| Ammonium sulphate | 130.4 |
| Magnesium sulphate heptahydrate | 121.5 |
| Manganese sulphate hydrate | 10 |
| Zinc sulphate hepthydrate | 2 |
| Copper sulphate pentahydrate | 0.025 |
| Calcium dihydrate | 113.2 |
| Potassium Iodide | 0.75 |
| Cobalt dichloride hexahydrate | 0.025 |
| Sodium phosphate monobasic dihydrate | 130.44 |
| Boric acid | 3 |
| Sodium molybdate dihydrate | 0.25 |
| FeNa - EDTA | 36.7 |
| Myo-inositol | 100 |
| Thiamine-HCL | 10 |
| Nicotinic acid | 1 |
| Pyridoxine-HCL | 1 |
| 2,4-D | 3 |
| Kinetin | 0.5 |
| Polyvinyl pyrrolidone (PVP10) | 1500 |
| Sucrose | 3 % |
